# *QuickStats:* Percentage Distribution of Deaths Attributed to Excessive Cold or Hypothermia,* by Month — United States, 2023

**DOI:** 10.15585/mmwr.mm7406a6

**Published:** 2025-02-27

**Figure:**
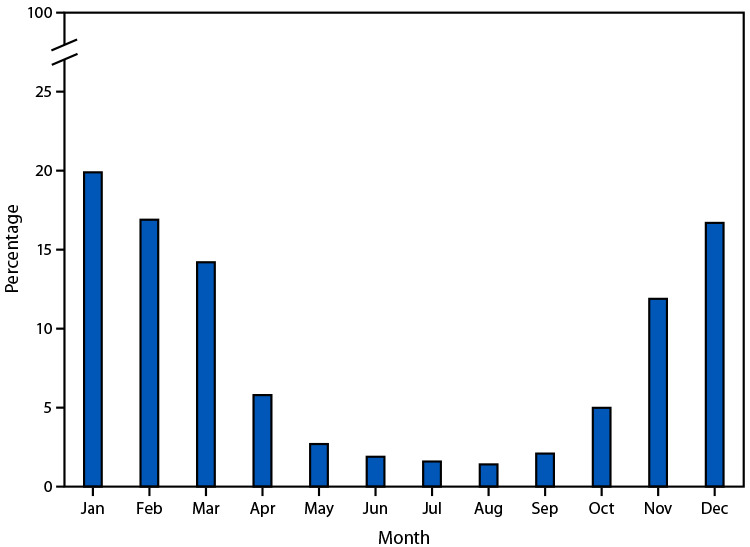
In 2023, a total of 1,024 deaths were attributed to excessive cold or hypothermia. The majority of deaths occurred during January–February and November–December, with the highest percentage occurring in January (19.9%).

**Supplementary Table:** https://stacks.cdc.gov/view/cdc/175597

**Source:** National Center for Health Statistics, National Vital Statistics System, Mortality Data, 2023. https://www.cdc.gov/nchs/nvss/deaths.htm

For more information on this topic, CDC recommends the following link: https://www.cdc.gov/winter-weather/prevention/index.html.

